# Urbanization in Peru is inversely associated with double burden of malnutrition: Pooled analysis of 92,841 mother–child pairs

**DOI:** 10.1002/oby.23188

**Published:** 2021-06-19

**Authors:** Daniel Mendoza‐Quispe, Akram Hernández‐Vásquez, J. Jaime Miranda, Cecilia Anza‐Ramirez, Rodrigo M. Carrillo‐Larco, Marco Pomati, Shailen Nandy, Antonio Bernabe‐Ortiz

**Affiliations:** ^1^ CRONICAS Center of Excellence in Chronic Diseases Universidad Peruana Cayetano Heredia Lima Peru; ^2^ School of Medicine Universidad Peruana Cayetano Heredia Lima Peru; ^3^ Department of Epidemiology and Biostatistics School of Public Health Imperial College London London UK; ^4^ School of Social Sciences Cardiff University Cardiff, Wales UK

## Abstract

**Objective:**

This study assessed the relationship between urbanization and the double burden of malnutrition (DBM) in Peru.

**Methods:**

A cross‐sectional analysis of the Demographic and Health Survey (2009 to 2016) was conducted. A DBM “case” comprised a child with undernutrition and a mother with overweight/obesity. For urbanization, three indicators were used: an eight‐category variable based on district‐level population density (inhabitants/km^2^), a dichotomous urban/rural variable, and place of residence (countryside, towns, small cities, or capital/large cities).

**Results:**

The prevalence of DBM was lower in urban than in rural areas (prevalence ratio [PR] 0.70; 95% CI: 0.65‐0.75), and compared with the countryside, DBM was less prevalent in towns (PR 0.75; 95% CI: 0.69‐0.82), small cities (PR 0.73; 95% CI: 0.67‐0.79), and capital/large cities (PR 0.53; 95% CI: 0.46‐0.61). Using population density, the adjusted prevalence of DBM was 9.7% (95% CI: 9.4%‐10.1%) in low‐density settings (1 to 500 inhabitants/km^2^), 5.9% (95% CI: 4.9%‐6.8%) in mid‐urbanized settings (1,001 to 2,500 inhabitants/km^2^), 5.8% (95% CI: 4.5%‐7.1%) in more densely populated settings (7,501 to 10,000 inhabitants/km^2^), and 5.5% (95% CI: 4.1%‐7.0%) in high‐density settings (>15,000 inhabitants/km^2^).

**Conclusions:**

The prevalence of DBM is higher in the least‐urbanized settings such as rural and peri‐urban areas, particularly those under 2,500 inhabitants/km^2^.

**Study Importance**

**What is already known?**

► In Peru, the coexistence of persistent child undernutrition and rising adult obesity rates drives the household‐level double burden of malnutrition (DBM), wherein children with undernutrition cohabit with adults with overweight/obesity.
► Increasing urbanization has been linked to DBM, despite half of the studies from published research finding nonsignificant associations. The simple urban versus rural area classification also fails to reflect important differences within these two categories.

**What does this study add?**

► This pooled cross‐sectional analysis of 92,841 mother–child pairs from the Demographic and Health Survey (2009 to 2016) shows that DBM in Peru is inversely associated with the degree of urbanization.
► The prevalence of DBM is greatest in the least‐urbanized settings, in rural and peri‐urban areas, and, in particular, in areas where population density is under 2,500 inhabitants/km^2^.
► Beyond this density level, the prevalence of DBM seems to remain lowest across more urbanized areas.

**How might these results change the direction of research?**

► Our findings may inform policies in Peru and similar countries, enabling them to be tailored and targeted at less urbanized settings, where the prevalence of DBM is highest.
► Further exploration of factors explaining changes in DBM in rural and peri‐urban areas could help with designing more effective interventions.
► This could provide the basis for similar research in other countries undergoing the nutrition transition, to see if this relationship is specific to Peru or more widely observable.

## INTRODUCTION

In Peru (1) and other low‐ and middle‐income countries (2, 3), the coexistence of child undernutrition (either underweight, wasting, and stunting) and rising adult obesity rates drives the household‐level double burden of malnutrition (DBM), wherein children with undernutrition paradoxically cohabit with mothers and other adults with overweight and/or obesity. In the 2009 to 2019 period in Peru, the prevalence of stunting among children under age 5 years fell from 23.8% to 12.2%; and concurrently, overweight/obesity among adult women of reproductive age (15 to 45 years) increased from 50.4% to 62.4% (4, 5). The DBM has emerged alongside rapid ongoing urbanization, particularly in developing regions where the latter process has evolved faster, exposing growing numbers of people to changing food systems that are typified by increased availability and consumption of “ultraprocessed” foods (6), paired with profiles of increased physical inactivity and sedentary lifestyles.

Increasing urbanization has been classically linked to DBM, despite roughly half of the studies finding nonsignificant associations (7). A common limitation has been a reliance on a simple dichotomous definition of urban or rural (7), and the evidence beyond this definition is scarce (8, 9). Such a classification, although useful, does not capture the complexity of nutrition dynamics and transition across different degrees of urbanization, which could be typified by levels of population density (10). By adopting a more nuanced measure of urbanization, we can explore the uneven characteristics within each stage of urbanization, such as people having different opportunities to access health care, education, improved sanitation, better incomes, and other factors determining nutrition and health outcomes (11).

In this paper, we use several categories of population density (10) to produce a more nuanced portrayal of where the Peruvian population lives, with each representing an increase in level of urbanization. We corroborate our findings using two additional indicators of urbanization: the conventional urban or rural definition and a four‐category definition comparing inhabitants from the countryside, towns, small cities, and capital/large cities. In short, this paper evaluates the association between degree of urbanization and DBM in Peru using anthropometric and household survey data from mother–child pairs.

## METHODS

### Study design

We performed a secondary analysis of two data sets merged using the district identifier: the Peruvian Demographic and Health Survey (DHS) (12) from 2009 to 2016 and a Peruvian geographic data set containing district‐level information (13), both collected by the National Institute of Statistics and Informatics (INEI, in Spanish). The Peruvian DHS is a nationwide survey, described elsewhere (14), with similar surveys run in almost 100 countries; the DHS is recognized for its high quality and usefulness in providing accurate and representative estimates regarding population, health, and nutrition (15). The Peruvian geographic data set included district population counts for each year analyzed and unique district surface areas in kilometers squared and altitude in meters above sea level (m.a.s.l.).

### Population, sample, and sampling

The Peruvian DHS data used in this paper were collected annually, using a two‐stage cluster random sampling approach and urban–rural stratification (14). In the first stage, clusters consisted of blocks agglomerating 120 to 140 households on average in urban areas and one or more villages together adding roughly 120 to 140 households in rural areas. Ten to fifteen households within corresponding clusters were selected in the second stage. As our interest is to assess DBM, all children under 5 years and their respective nonpregnant mothers (aged 15 to 49 years) living within the same household were included. Each child was paired with his or her mother and thus these are referred to as mother–child pairs; cases in which two (*n* = 22,170), three (*n* = 2,220), or four (*n* = 56) children lived with the same mother were evaluated as separate, distinct mother–child pairs. Mother–child pairs in which children had extreme (i.e., improbable) anthropometric measurements and those in which mothers were not identified or had incomplete anthropometrics were excluded.

### Anthropometric measurements

Trained fieldworkers applied health and demographic questionnaires through direct interviews at households of eligible participants. Afterward, participants underwent anthropometric measurement by qualified anthropometrists (14). For weight determinations, Seca electronic scales (Hamburg, Germany) with a precision of 50 g (Model 872) or 100 g (Model 881) were used for both children and adults. Heights of children under 2 years were measured in a lying position using an "infantometer," and weights were taken in a standing position using the difference in kilograms of the mother with and without the child. The heights of children aged 2 to 5 years were measured using a stadiometer, and their weights were measured in standing position. Adult measurements were taken in a standing position. Instruments were periodically assessed for quality control. When scheduled, cross validation of measures between the local supervisor and the anthropometrist was conducted.

### Outcome variable

The DBM was the outcome of interest; its two components (child undernutrition and maternal overweight/obesity) were also evaluated. The DBM comprised the coexistence of a child with undernutrition and the corresponding mother with overweight/obesity. A child was considered undernourished if he or she was affected by either stunting, underweight, or wasting. We used height‐for‐age *z* scores to define stunting, weight‐for‐age *z* scores for underweight, and weight‐for‐height *z* scores for wasting, using the standard threshold of <−2 SDs from the World Health Organization (WHO) 2006 international child growth standards (16). Children with extreme measurements (<−5 SDs, >+5 SDs) were excluded, following WHO guidelines (17). The rationale behind using the compound outcome comes from the Composite Index of Anthropometric Failure, which reflects the increased morbidity and mortality in children with multiple deficits, and was designed to challenge the underestimation of malnutrition when only one indicator is used (18, 19). Maternal overweight/obesity was assessed using BMI, according to the standard cutoff *(*BMI ≥25 kg/m^2^) (20). For descriptive analyses, outcomes were disaggregated as follows: mother–child pairs were classified as “normal only,” “child undernutrition only,” “maternal overweight/obesity only,” and “DBM” (i.e., a child with undernutrition and a mother with overweight/obesity) (7). Undernutrition in children was based on their experience of any form of undernutrition as identified by the Composite Index of Anthropometric Failure (18), and for mothers, the standard categories (20) normal (BMI <25 kg/m^2^), overweight (BMI ≥25 kg/m^2^ and BMI <30 kg/m^2^), and obesity (BMI ≥30 kg/m^2^) were presented separately.

### Exposure variable

Urbanization conceptually means changes in the size, density, and built‐in characteristics of cities, although the classification of what is urban differs across countries and studies (21). In this paper, district‐level population density was used as an indicator of urbanization level. First, population density in inhabitants/km^2^ (inh/km^2^) was calculated by dividing the number of district inhabitants in the specific year by the district surface area in square kilometers. Population counts were generated by INEI following the methodology of the United Nations to produce population estimates and projections (22). Based on the population density component from a validated urbanicity scale (10), the following eight ascending categories were defined: 1 to 500; 501 to 1,000; 1,001 to 2,500; 2,501 to 5,000; 5,001 to 7,500; 7,501 to 10,000; 10,001 to 15,000; and ≥15,001 inh/km^2^. The first, middle, and last categories theoretically represent low‐, mid‐, and highly urbanized areas, respectively. For sensitivity analyses, two alternative definitions provided in DHS data were considered: (1) the usual urban–rural definition, in which urban areas were made up of streets and blocks with grouped households and ≥2,000 inhabitants and rural areas that had <2,000 inhabitants and scattered housing; and (2) a four‐category definition that grouped inhabitants from the countryside (rural areas), towns, small cities (more than 50,000 inhabitants), and capital/large cities (more than 1 million inhabitants) (23).

### Covariates

Relevant covariates were selected a priori according to existent literature (7) and used to adjust regression models. These were child’s sex (female, male), child's age (<2, 2 to 5 years), mother’s age (15 to 24, 25 to 34, 35 to 49 years), mother’s highest educational attainment (primary or less, secondary, superior), household socioeconomic status in quintiles (very poor, poor, middle, rich, very rich), and district‐level altitude (<2,500, ≥2,500 m.a.s.l.). Socioeconomic status was derived from a wealth index score (provided in DHS data) based on household assets (24), split in quintiles separately for urban and rural areas, and then combined (25).

### Data analysis

Population characteristics were described using unweighted frequencies and survey‐weighted percentages. To provide context about the overall trajectory of the outcomes during the study period, we briefly report temporal trends of DBM and its components between 2009 and 2016 in online Supporting Information, evaluated by regressing each category of the outcomes on the survey year in Poisson log models and reporting annual prevalence along with 2016 to 2009 differences. Then, in the pooled data set, Poisson log generalized linear regression models were fitted to evaluate the association between urbanization level and DBM. The model was adjusted by the aforementioned covariates, introducing the survey year as a continuous variable and taking the least‐urbanized group as reference. The variance inflation factor (VIF) was used to verify collinearity between independent variables; however, none exhibited high collinearity (all VIF estimates were under 3). Prevalence ratios (PR) and 95% CI are reported. In addition, we plotted estimates on predicted crude and adjusted prevalence of the outcome by urbanization level. To improve the understanding of differences across adjacent urbanization levels (rather than comparing all with one reference group), Bonferroni‐adjusted (considering *p* < 0.05/7 comparisons = *p* < 0.007) post hoc pairwise comparisons using contrasts of marginal linear predictions were evaluated between a specified urbanization level against the upper‐immediate level (e.g., 1,000 to 2,500 inh/km^2^ as reference vs. 2,501 to 5,000 inh/km^2^) and also between the balanced prevalence of previous levels against each urbanization level (e.g., <2,501 inh/km^2^ as reference vs. 2,501 to 5,000 inh/km^2^). The *p* value of global significance (multidegree of freedom test) of the urbanization variable was also reported. A sensitivity analysis was conducted using the two other definitions of urbanization described here, following the same approach. To assess whether short maternal height is associated with chronic undernutrition earlier in life or with current maternal overweight or obesity, the mother's height variable was regressed against child undernutrition and, separately, mother’s overweight/obesity in the overall sample, and then the analysis was stratified by place of residence. Lastly, in order to evaluate whether the aggregated prevalence of DBM across urbanization levels is independent of its components (*a*, child undernutrition; *b*, maternal overweight/obesity), the expected prevalence of DBM (a*b/100, i.e., the product of the aggregated prevalence of child undernutrition and the prevalence of maternal overweight/obesity divided by 100) and the ratio between *c* (DBM) and each component (*c*/*a*, and *c*/*b*) were calculated. All analyses were conducted in Stata Statistical Software version 15.0 (StataCorp LLC), using the *svy* command to account for the complex survey design, and graphs were designed in R using the *ggplot2* library. Statistical significance was evaluated at *p* < 0.05, except for the Bonferroni‐adjusted analysis, as indicated here previously.

### Ethics

Both the DHS and the geographic data sets containing anonymized data were retrieved from open access websites (https://www.inei.gob.pe/ and http://institutodelperu.pe/) (12, 13). The study protocol of this secondary analysis was approved by the Ethics Committee of the Universidad Peruana Cayetano Heredia (SIDISI 200933).

## RESULTS

### Population description

**FIGURE 1:**
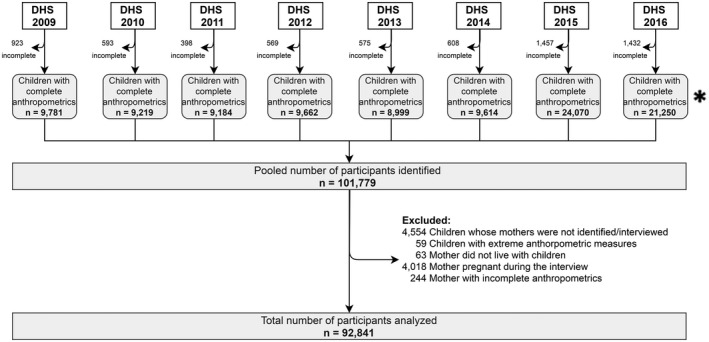
Flowchart of the sample. *Initial sample equaled official population estimates reported by INEI in Peru (14). DHS, Demographic and Health Survey; INEI, National Institute of Statistics and Informatics

Data from 92,841 mother–child pairs were analyzed (Figure 1) after excluding 8,938 pairs (8.8%), of whom 4,554 (51%) mothers were not identified and 4,018 were pregnant (45%) at the time of the survey. Characteristics of included and excluded participants were similar, except that a higher proportion of excluded children were 2 to 5 years old compared with the included ones (78% vs. 58%, Supporting Information Table S1). The evaluation of trends in DBM between 2009 and 2016 is provided in Supporting Information Table S2.

In the pooled population, the prevalence of maternal overweight/obesity, child undernutrition, and DBM were 59.7% (95% CI: 59.1%‐60.3%), 18% (95% CI: 17.6%‐18.5%), and 9% (95% CI: 8.7%‐9.3%), respectively (Supporting Information Table S3). Children experiencing “only stunting” (14.4%) composed the bulk of undernutrition (Supporting Information Table S2), followed by those who were simultaneously affected by “stunting” and “underweight*”* (2.8%). The remaining combinations of undernutrition amounted to roughly 1%. The study sample had 61.7% of the participants living in areas below 500 inh/km^2^, and the rest were distributed in the remaining categories. Bivariate analysis shows that children were more likely to be in a DBM household if male, to be aged 2 to 5 years, to have older mothers and mothers with no/low education, to be living in households in lower wealth quintiles, and to be living at high altitude (≥2,500 m.a.s.l.) (Supporting Information Table S4).

### Association between urbanization level and DBM components

**FIGURE 2:**
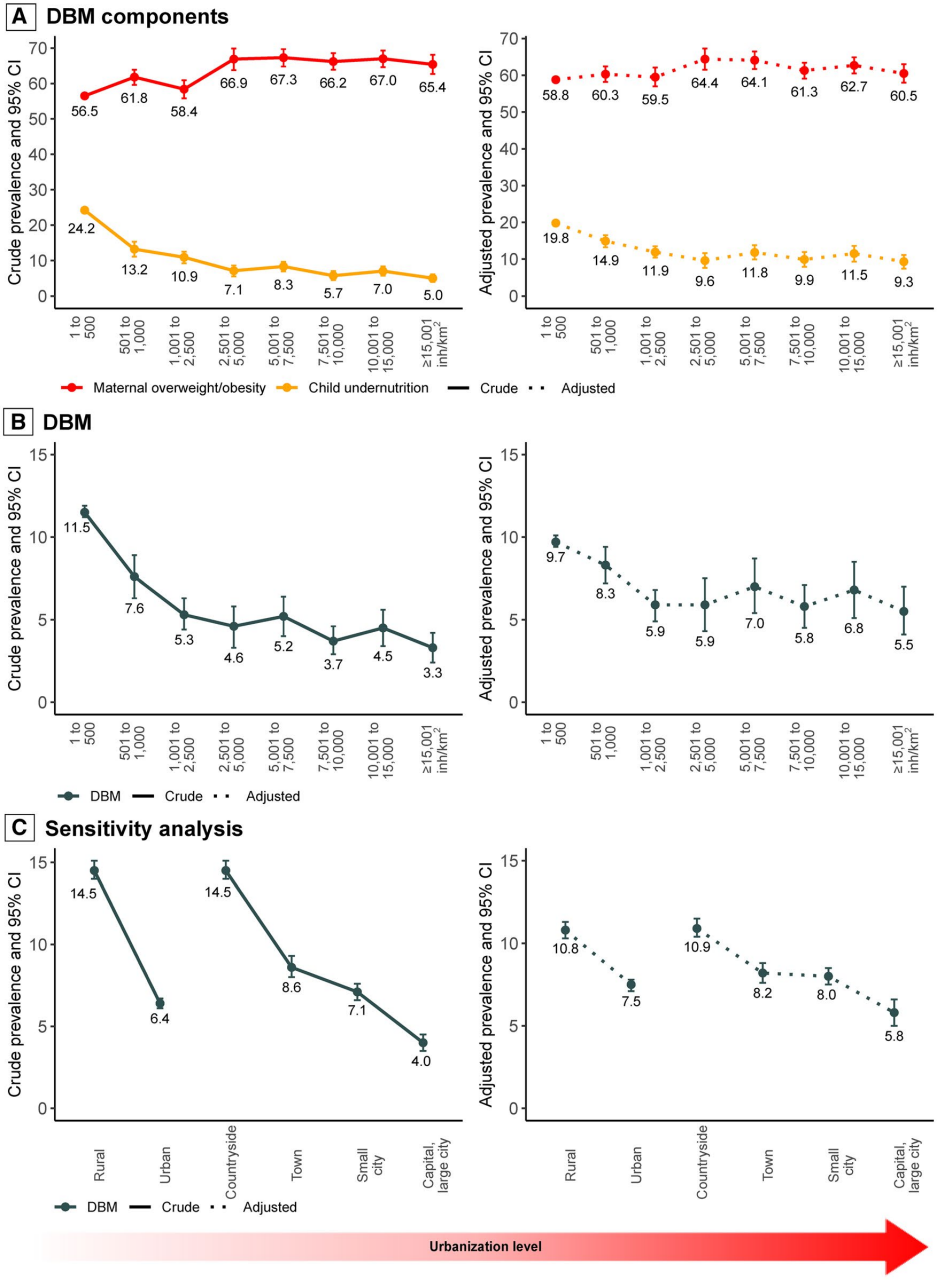
Prevalence of household‐level DBM and its components by urbanization level. Adjusted by urbanization level, child sex and age, mother’s age and highest educational attainment, socioeconomic status, altitude, and survey year. Full models shown in Table 1 and Table 2. DBM, double burden of malnutrition

In an unadjusted analysis (Figure 2A), the crude prevalence of child undernutrition declined nonlinearly (global *p* value of the urbanization variable <0.001) in areas with higher levels of urbanization. Conversely, the curve of maternal overweight/obesity illustrated a direct relationship with urbanization (global *p* value of the urbanization variable <0.001). The adjusted curve (dotted lines) confirmed the nonlinear inverse relationship between urbanization and child undernutrition, although above 2,500 inh/km^2^, on average, the plot is almost flat. In contrast, the adjusted plot of maternal overweight/obesity was almost flat across the whole urbanization gradient. Table 1 shows the percentage difference in the prevalence of each DBM component between the least (1 to 500 inh/km^2^) and highest (≥15,001 inh/km^2^) urbanized areas. Although the differences in the likelihood of maternal overweight/obesity between these two areas is, on average, small (PR 1.03; 95% CI: 0.98‐1.07), it is considerably greater for the prevalence of child undernutrition (which is 53% significantly lower in the highest‐urbanized areas; PR 0.47; 95% CI: 0.38‐0.5).

**TABLE 1:** Association between urbanization and household‐level DBM components

	Crude prevalence		Crude model^a^		Adjusted model^a^	
	%	95% CI	PR	95% CI	PR	95% CI
(A) Child undernutrition						
Urbanization level (inh/km^2^)						
1 to 500	24.2	23.6‐24.9	Ref.		Ref.	
501 to 1,000	13.2	11.1‐15.3	**0.54**	**0.46‐0.64**	**0.75**	**0.67‐0.84**
1,001 to 2,500	10.9	9.2‐12.5	**0.45**	**0.38‐0.52**	**0.60**	**0.53‐0.69**
2,501 to 5,000	7.1	5.5‐8.6	**0.29**	**0.23‐0.36**	**0.48**	**0.39‐0.60**
5,001 to 7,500	8.3	6.9‐9.6	**0.34**	**0.29‐0.40**	**0.60**	**0.51‐0.70**
7,501 to 10,000	5.7	4.5‐7	**0.24**	**0.19‐0.30**	**0.50**	**0.41‐0.61**
10,001 to 15,000	7.0	5.7‐8.3	**0.29**	**0.24‐0.35**	**0.58**	**0.48‐0.70**
≥15,001	5.0	4‐6.1	**0.21**	**0.17‐0.26**	**0.47**	**0.38‐0.57**
(B) Maternal overweight/obesity						
Urbanization level (inh/km^2^)						
1 to 500	56.5	55.9‐57.2	Ref.		Ref.	
501 to 1,000	61.8	59.6‐63.9	**1.09**	**1.05‐1.13**	1.02	0.99‐1.06
1,001 to 2,500	58.4	55.8‐60.9	1.03	0.99‐1.08	1.01	0.97‐1.06
2,501 to 5,000	66.9	63.8‐69.9	**1.18**	**1.13‐1.24**	**1.09**	**1.04‐1.15**
5,001 to 7,500	67.3	64.8‐69.7	**1.19**	**1.14‐1.24**	**1.09**	**1.05‐1.13**
7,501 to 10,000	66.2	63.9‐68.6	**1.17**	**1.13‐1.22**	**1.04**	**1.00‐1.08**
10,001 to 15,000	67.0	64.6‐69.3	**1.18**	**1.14‐1.23**	**1.07**	**1.03‐1.11**
≥15,001	65.4	62.7‐68.1	**1.16**	**1.11‐1.21**	1.03	0.98‐1.07

Model adjusted by urbanization level, sex and age of child, age and educational attainment of mother, socioeconomic status, altitude, and survey year.

Abbreviations: DBM, double burden of malnutrition; inh/km^2^, inhabitants/km^2^; PR, prevalence ratio; Ref., reference.

^a^ Poisson log generalized linear models, accounting for the complex survey design.

Estimates with *p* < 0.05 shown in bold.

In addition, there was no evidence of a difference on overweight/obesity status among mothers with short height from those with normal height. However, children were twice more likely to be undernourished when mothers had short height, compared with children of mothers with normal height. This relationship was stronger in urban (PR 2.88; 95% CI: 2.67‐3.12) than in rural areas (PR 1.90; 95% CI: 1.83‐1.98; Supporting Information Table S5).

### Association between urbanization level and DBM

Figure 2B shows that the unadjusted rate of DBM declines at higher levels of urbanization (global *p* value of the urbanization variable < 0.001) resembling a linear pattern from 11.5% (95% CI: 11.2%‐11.9%) to 5.3% (95% CI: 4.4%‐6.3%) up to 2,500 inh/km^2^ and then falling monotonically to 3.3% (95% CI: 2.4%‐4.2%) for the most urbanized settings (≥15,001 inh/km^2^). In the adjusted analysis, we also found that as urbanization level increases, the adjusted prevalence of DBM declines (global *p* < 0.001) also from low‐ (9.7%; 95% CI: 9.4%‐10.1%) to mid‐urbanized settings (5.9%; 95% CI: 4.9%‐6.8%) up to an urban density of 2,500 inh/km^2^ on average. However, beyond this point, the association between urbanization and DBM plateaus. Together, these findings suggest that DBM in Peru is higher in the least‐urbanized settings, particularly those under 2,500 inh/km^2^, and areas with urbanization beyond this level have similar low prevalence of DBM.

Table 2 shows that the adjusted prevalence of DBM in the highest‐urbanized areas (≥15,001 inh/km^2^) was 43% lower (PR 0.57; 95% CI: 0.44‐0.74) compared with the least‐urbanized ones (1 to 500 inh/km^2^). Table 3 corroborates the findings further by first comparing each level of urbanization with the next one (e.g., 1 to 500 with 501 to 1,000, then 501 to 1,000 with 2,501 to 5,000) as well as comparing a given level with all lower levels (e.g., 501 to 1,000 with <500). Overall, we find statistically significant decreases in adjusted DBM rates from the lowest level of urbanization up to 1,001 to 2,500 inh/km^2^ but not beyond this point.

**TABLE 2:** Association between urbanization and household‐level DBM

Exposure	Crude prevalence		Crude model^a^		Adjusted model 1^a^		Adjusted model 2^a^		Adjusted model 3^a^	
	%	95% CI	PR	95% CI	PR	95% CI	PR	95% CI	PR	95% CI
Urbanization level (inh/km^2^)										
1 to 500	11.5	11.2‐11.9	Ref.		Ref.		Ref.		Ref.	
501 to 1,000	7.6	6.3‐8.9	**0.66**	**0.55‐0.78**	**0.82**	**0.70‐0.97**	**0.76**	**0.65‐0.88**	**0.85**	**0.74‐0.98**
1,001 to 2,500	5.3	4.4‐6.3	**0.46**	**0.39‐0.55**	**0.65**	**0.55‐0.77**	**0.47**	**0.40‐0.56**	**0.60**	**0.51‐0.71**
2,501 to 5,000	4.6	3.3‐5.8	**0.39**	**0.30‐0.52**	**0.52**	**0.39‐0.69**	**0.53**	**0.40‐0.70**	**0.61**	**0.46‐0.80**
5,001 to 7,500	5.2	4.0‐6.4	**0.45**	**0.36‐0.57**	**0.60**	**0.48‐0.76**	**0.64**	**0.51‐0.80**	**0.72**	**0.57‐0.91**
7,501 to 10,000	3.7	2.9‐4.6	**0.32**	**0.26‐0.41**	**0.48**	**0.38‐0.61**	**0.52**	**0.41‐0.65**	**0.60**	**0.47‐0.75**
10,001 to 15,000	4.5	3.4‐5.6	**0.39**	**0.31‐0.50**	**0.57**	**0.44‐0.73**	**0.60**	**0.47‐0.78**	**0.70**	**0.55‐0.90**
≥15,001	3.3	2.4‐4.2	**0.29**	**0.22‐0.38**	**0.44**	**0.33‐0.57**	**0.49**	**0.38‐0.64**	**0.57**	**0.44‐0.74**
*N* (unweighted)			92,208		92,207		90,247		90,246	

Model 1 adjusted by urbanization level, child sex and age, mother’s age and highest educational attainment, and survey year.

Model 2 adjusted by urbanization level, socioeconomic status, altitude, and survey year.

Model 3 adjusted by urbanization level, child sex and age, mother’s age and highest educational attainment, socioeconomic status, altitude, and survey year.

Abbreviations: DBM, double burden of malnutrition; inh/km^2^, inhabitants/km^2^; *N*, number of observations included in the model; PR, prevalence ratio; Ref., reference.

^a^ Poisson log generalized linear models, accounting for the complex survey design. Estimates with *p* < 0.05 shown in bold.

**TABLE 3:** Comparison of the prevalence of DBM between a specified urbanization level and the upper‐immediate level

Exposure	Crude model^a^		Adjusted model^a^	
	PR	95% CI	PR	95% CI
Urbanization level (inh/km^2^)				
1 to 500 (ref.) vs. 501 to 1,000	**0.66**	**0.51‐0.84**	0.85	0.70‐1.03
501 to 1,000 (ref.) vs. 1,001 to 2,500	0.70	0.50‐0.99	0.71	0.53‐0.95
1,001 to 2,500 (ref.) vs. 2,501 to 5,000	0.86	0.55‐1.34	1.01	0.65‐1.56
2,501 to 5,000 (ref.) vs. 5,001 to 7,500	1.15	0.70‐1.87	1.19	0.73‐1.93
5,001 to 7,500 (ref.) vs. 7,501 to 10,000	0.72	0.45‐1.13	0.83	0.53‐1.30
7,501 to 10,000 (ref.) vs. 10,001 to 15,000	1.21	0.76‐1.93	1.17	0.74‐1.85
10,001 to 15,000 (ref.) vs. ≥15,001	0.74	0.45‐1.21	0.81	0.50‐1.31
<500 (ref.) vs. 501 to 1,000	**0.66**	**0.51‐0.84**	0.85	0.70‐1.03
<1,001 (ref.) vs. 1,001 to 2,500	**0.57**	**0.44‐0.74**	**0.66**	**0.51‐0.84**
<2,501 (ref.) vs. 2,501 to 5,000	**0.59**	**0.40‐0.87**	0.76	0.52‐1.12
<5,001 (ref.) vs. 5,001 to 7,500	0.77	0.55‐1.08	0.97	0.69‐1.35
<7,501 (ref.) vs. 7,501 to 10,000	**0.58**	**0.41‐0.82**	0.80	0.58‐1.12
<10,001 (ref.) vs. 10,001 to 15,000	0.77	0.54‐1.10	0.98	0.69‐1.40
<15,001 (ref.) vs. ≥15,001	**0.59**	**0.40‐0.86**	0.80	0.55‐1.15
Global *p* value	**<0.001**		**<0.001**	

Estimates with Bonferroni‐adjusted *p* < 0.007 shown in bold.

Full model adjusted by urbanization level, sex and age of child, age and educational attainment of mother, socioeconomic status, altitude, and survey year.

Abbreviations: DBM, double burden of malnutrition; inh/km^2^, inhabitants/km^2^; PR, prevalence ratio.

^a^ Contrasts of marginal linear predictions from Poisson log generalized linear models, with Bonferroni correction for multiple comparisons, and accounting for the complex survey design.

In Table 4, (1) urbanization was inversely correlated with both child undernutrition and mothers’ overweight/obesity, (2) maternal overweight/obesity do not show this relative difference between high‐ and low‐density areas, and (3) the expected prevalence of DBM matched the observed prevalence across urbanization categories.

**TABLE 4:** Relationship between the aggregated prevalence of child undernutrition, maternal overweight/obesity, and DBM

Exposure	Observed crude prevalence^a^ (%)			DBM expected prevalence (A*B/100)	C/A ratio	C/B ratio
	(A) Child undernutrition	(B) Maternal overweight/obesity	(C) DBM			
Urbanization level (inh/km^2^)						
1 to 500	24.2	56.5	11.5	13.7	0.48	0.20
501 to 1,000	13.2	61.8	7.6	8.2	0.58	0.12
1,001 to 2,500	10.9	58.4	5.3	6.4	0.49	0.09
2,501 to 5,000	7.1	66.9	4.6	4.7	0.65	0.07
5,001 to 7,500	8.3	67.3	5.2	5.6	0.63	0.08
7,501 to 10,000	5.7	66.2	3.7	3.8	0.65	0.06
10,001 to 15,000	7.0	67.0	4.5	4.7	0.64	0.07
≥15,001	5.0	65.4	3.3	3.3	0.66	0.05

Exposure	Observed adjusted prevalence^a^ (%)			DBM expected prevalence (A*B/100)	C/A ratio	C/B ratio
	(A) Child undernutrition	(B) Maternal overweight/obesity	(C) DBM			
Urbanization level (inh/km^2^)						
1 to 500	19.8	58.8	9.7	11.6	0.49	0.16
501 to 1,000	14.9	60.3	8.3	9.0	0.56	0.14
1,001 to 2,500	11.9	59.5	5.9	7.1	0.50	0.10
2,501 to 5,000	9.6	64.4	5.9	6.2	0.61	0.09
5,001 to 7,500	11.8	64.1	7.0	7.6	0.59	0.11
7,501 to 10,000	9.9	61.3	5.8	6.1	0.59	0.09
10,001 to 15,000	11.5	62.7	6.8	7.2	0.59	0.11
≥15,001	9.3	60.5	5.5	5.6	0.59	0.09

Model adjusted by urbanization level, sex and age of child, age and educational attainment of mother, socioeconomic status, altitude, and survey year.

Abbreviations: DBM, double burden of malnutrition; inh/km^2^, inhabitants/km^2^.

^a^ Poisson log generalized linear models, accounting for the complex survey design.

### Sensitivity analysis

The nonlinear negative relationship between urbanization and DBM was confirmed in our sensitivity analyses using additional categories of urbanization (Table 5). Using the urban–rural dichotomy, the adjusted prevalence of DBM was 30% lower in urban than in rural areas (PR 0.70; 95% CI: 0.65‐0.75). Using the four‐category definition, compared with the countryside (least‐urbanized), DBM was less widespread in towns (adjusted PR 0.75; 95% CI: 0.69‐0.82), small cities (adjusted PR 0.73; 95% CI: 0.67‐0.79), and capital/large cities (adjusted PR 0.53; 95% CI: 0.46‐0.61). However, using the measure of urbanization with several categories of population density in the main analysis, compared with both alternative definitions, we were able to discern that the inverse relationship between urbanization and DBM plateaus beyond the urban density of 2,500 inh/km^2^ (Figure 2C vs. 2B).

**TABLE 5:** Urbanization and household‐level DBM: sensitivity analysis

Exposures	Crude prevalence		Crude model^a^		Adjusted model 1^a^		Adjusted model 2^a^		Adjusted model 3^a^	
	%	95% CI	PR	95% CI	PR	95% CI	PR	95% CI	PR	95% CI
Urban–rural dichotomy										
Rural	14.5	14‐15.1	Ref.		Ref.		Ref.		Ref.	
Urban	6.4	6.1‐6.7	**0.44**	**0.41‐0.47**	**0.68**	**0.63‐0.73**	**0.53**	**0.49‐0.57**	**0.70**	**0.65‐0.75**
Four‐categories definition										
Countryside	14.5	14‐15.1	Ref.		Ref.		Ref.		Ref.	
Town	8.6	8.0‐9.3	**0.59**	**0.54‐0.65**	**0.82**	**0.75‐0.89**	**0.59**	**0.54‐0.64**	**0.75**	**0.69‐0.82**
Small city	7.1	6.6‐7.6	**0.49**	**0.45‐0.53**	**0.73**	**0.67‐0.79**	**0.55**	**0.51‐0.60**	**0.73**	**0.67‐0.79**
Capital, large city	4.0	3.5‐4.5	**0.27**	**0.24‐0.31**	**0.44**	**0.38‐0.51**	**0.41**	**0.35‐0.47**	**0.53**	**0.46‐0.61**
*N* (unweighted)			92,841		92,840		90,684		90,683	

Model 1 adjusted by urbanization level, child sex and age, mother’s age and highest educational attainment, and survey year.

Model 2 adjusted by urbanization level, socioeconomic status, altitude, and survey year.

Model 3 adjusted by urbanization level, child sex and age, mother’s age and highest educational attainment, socioeconomic status, altitude, and survey year.

Abbreviations: DBM, double burden of malnutrition; *N* , number of observations included in the model; PR, prevalence ratio; Ref., reference.

^a^ Poisson log generalized linear models, accounting for the complex survey design. Estimates with *p* < 0.05 shown in bold.

## DISCUSSION

Urbanization in Peru is inversely associated with household‐level DBM, regardless of using a nuanced or less‐nuanced measure of urbanization. That said, DBM is higher in the least‐urbanized settings such as rural and peri‐urban areas, particularly those under 2,500 inh/km^2^ on average. Beyond this density level, the prevalence of DBM seems to remain lowest across more urbanized areas. By studying a variety and more nuanced definitions of urbanization, we provide insights into the evolving relationship between urbanization and DBM in Peru and similar countries. Results from our study can inform and shape relevant policies to guarantee high‐quality nutrition and child development strategies in rural remote areas.

Higher DBM rates in rural areas can be explained in several ways. Urbanization is no longer a phenomenon exclusive to large cities; nowadays its consequences rebound across rural landscapes, exemplified by the “urbanization of rural life” but without accompanying infrastructure of roads and streets and instead characterized by changing dietary and physical activity patterns (26). In the past, a significant proportion of rural inhabitants in many countries in the region relied on their own home production of food; now for many, their diets consist mainly of market‐purchased, processed products (27). Furthermore, the combination of (1) a rapid expansion of small supermarkets, with marked penetration of snacks and ultraprocessed products (27), (2) lower prices for processed foods resulting in greater accessibility to the poorest sections of society (11), and (3) changes in food quality, including the replacement of water by sweetened beverages, lower fruits and vegetables intake, and their replacement by energy‐dense foods in larger portion sizes (26, 28), have accentuated rural food insecurity. Ultraprocessed foods in early life may also contribute to child stunting (3).

All aforementioned behavioral changes and obesogenic environments (7, 29) explain faster BMI increases among adult women in rural areas (30, 31, 32). In this line, although adult female overweight/obesity seems to be the main driver of DBM (33), child undernutrition rates remain persistent and higher in rural areas (34), and thus, rural households will experience DBM. Furthermore, despite the well‐documented growing burden of obesity in Peru, our results show that globally and at every level of urbanization, DBM depends on the combination of both child undernutrition and maternal overweight/obesity. Hence, from a policy perspective, double‐duty actions focusing on ending child undernutrition in all its forms and reducing maternal overweight/obesity remain equally important for addressing DBM.

On the international side (7, 8, 9, 31, 35), data suggest intercountry variation in the prevalence of DBM between urban and rural areas, and so directionality of the association may vary depending on what stage of the nutrition transition a country is at (3, 36). In a systematic review from 2017, over half the studies reviewed (22/41 = 54%) reported a positive relationship between urbanization and DBM; one of them was negative, but the rest were nonsignificant (7). Our results are similar to those from Colombia and Mexico in terms of finding an inverse relationship between urbanization and DBM (31, 35). In‐depth explorations beyond the urban–rural dichotomy, using cluster‐level population densities, point toward peri‐urban areas: compared with rural counterparts, the odds of DBM were higher among peri‐urban households in sub‐Saharan Africa (odds ratio 1.24; 95% CI: 1.06‐1.44) and Bolivia (odds ratio 1.8; 95% CI: 1.2‐2.7), although urban residency was directly associated with DBM only in sub‐Saharan Africa (odds ratio 1.24; 95% CI: 1.06‐1.46) (8, 9).

The DBM imposes additional short‐ and long‐term penalties: early onset malnutrition is difficult to offset after infancy; child stunting and maternal obesity exhibit intergenerational transmission; and children with undernutrition have compromised responses to infectious disease, impaired cognitive development, and a predisposition to obesity (37). The “capacity‐load” model of nutrition dynamics poses that early undernutrition reduces the metabolic capacity later into adolescence and adulthood, compromising the ability to maintain healthy blood pressure, weight, and glucose levels, which in turn can lead to the onset of cardiovascular diseases at younger ages (11, 37). Following this rationale, rural women affected by stunting during infancy and childhood may well be today’s mothers with overweight/obesity. Our findings also showed an association between short maternal height and child undernutrition, which was stronger in urban than in rural areas.

Tackling DBM requires dual action efforts both reducing undernutrition and preventing obesity, including nutrition strategies addressing the social and economic determinants of health, i.e., access to quality food, sanitation, housing, health services, and education (2). Further exploration of factors explaining changes in DBM in rural and peri‐urban areas, at or below the 2,500 inh/km^2^ threshold in Peru, could help with designing and targeting more effective interventions.

**FIGURE 3:**
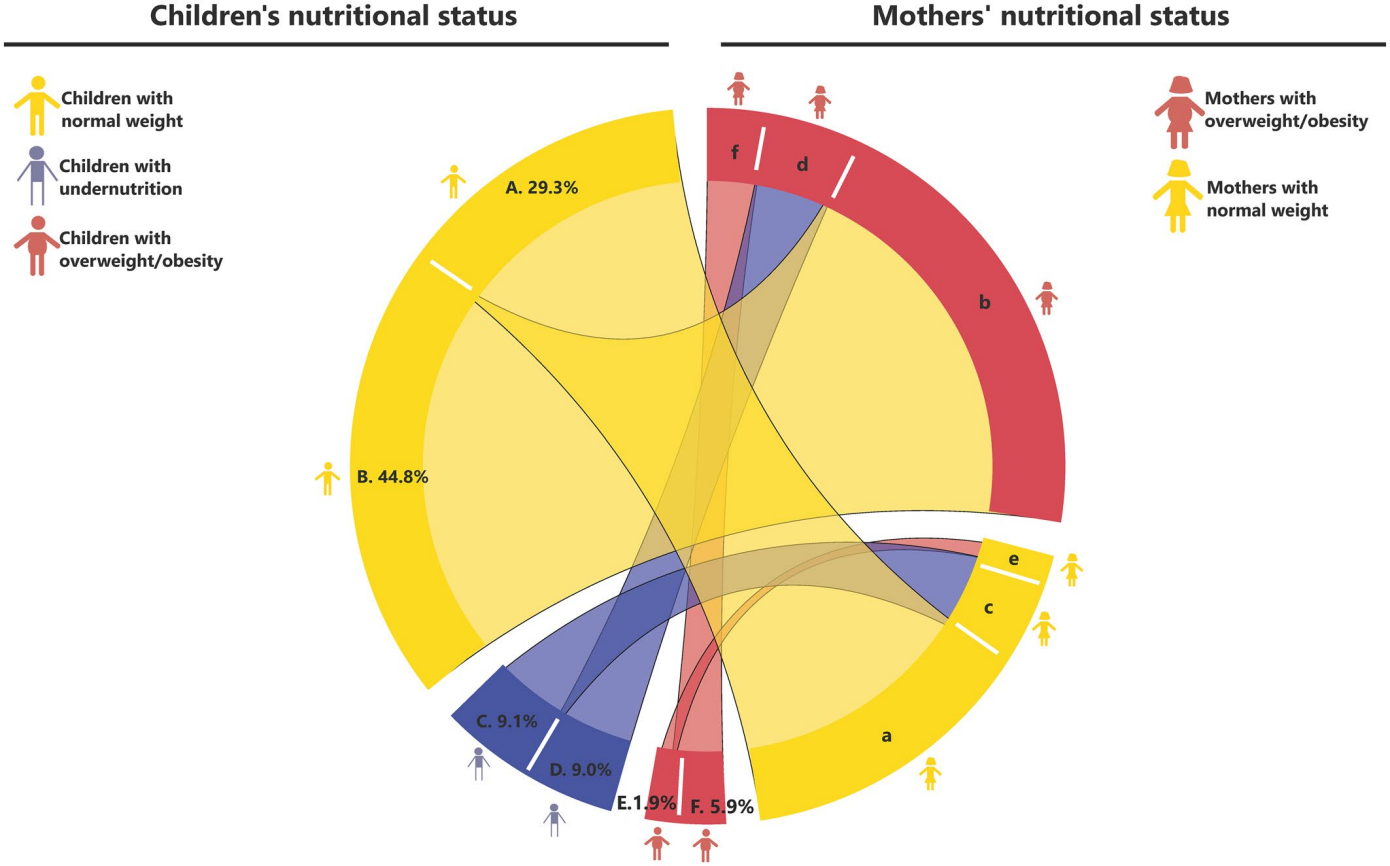
Nutritional status combinations between mothers and children. Percentages shown represent the proportion of children under 5 in the sample who live with mothers in a given pattern of nutritional status. For example, section “A” shows that 29.3% of children classed as having normal weight (section “A”) live with mothers who also have “normal” weight (section “a”). The percentages shown in the children categories sum to 100%

Regarding the strengths of the study, this large‐sample pooled analysis yielded more than 90,000 mother–child pairs from a nationally representative survey. Population density in this research was a simple but powerful indicator of the level of urbanization, able to distinguish small changes in Peru’s nutrition dynamics. Our study also had some limitations. First, the definition of the household‐level DBM may vary across studies, given the many possible combinations of the children and mothers’ nutritional categories, as shown in Figure 3. In this paper, we choose the most commonly used combination of children with undernutrition and mothers with overweight/obesity (7), with an overall prevalence of 9%. Other combinations, such as both children and mothers with overweight/obesity, had a prevalence of 5.9% and were not the focus of our research question. Second, we did not have information about food intake and physical activity, a limitation of using DHS data to examine nutrition dynamics (7). Other factors being recently explored such as breastfeeding (38, 39), parental height (40), or child prematurity also merit attention. Data on other district‐level features (10), such as number of supermarkets, other indicators of obesogenic environments, or broader measures of human development were also unavailable. Third, children excluded from the analysis had a higher proportion that were aged 2 to 5 years compared with the included ones (78% vs. 58%), because children were older in those pairs whose mothers were currently pregnant, and these were excluded. Although this could affect our estimates of DBM given that stunting is more visible in older young children, the 2 to 5‐year‐old age group, in children included (i.e., analyzed), was similar to the whole sample (58.6% vs. 60.3%), reducing selection bias. Finally, our results stem from mothers of childbearing age and preschoolers and do not consider other age range sets or fathers with overweight or obesity.

## CONCLUSION

Urbanization in Peru is inversely associated with household‐level DBM, regardless of using a nuanced or less‐nuanced measure of urbanization. In order to protect future generations from the negative outcomes linked to poor nutrition and hampered development in infancy and to reduce harmful effects of obesity in adults, our findings suggest the need to tailor and prioritize double‐duty strategies against DBM in the least‐urbanized, i.e., rural and peri‐urban, settings in Peru.

## CONFLICT OF INTEREST

The authors declared no conflict of interest.

## AUTHOR CONTRIBUTIONS

DM‐Q, AH‐V, CA‐R, RMC‐L, MP, SN, JJM, and AB‐O participated in the conception and design of this study, revised the manuscript, critically reviewed it, and approved the final version of this study. DM‐Q wrote the first draft of this manuscript. DM‐Q, AH‐V, and AB‐O participated in the acquisition of data sets and conducted the analysis of this study.

## Supporting information

Supplementary Material
