# Supplementary material for: Urbanization in Peru is inversely associated with double burden of malnutrition: Pooled analysis of 92,841 mother–child pairs
Source: Obesity (Silver Spring). 2021 Jun 19;29(8):1363–74. doi: 10.1002/oby.23188 (PMC8361670; doi:10.1002/oby.23188)
Supplement: Supplementary file 1 — Supplementary Material [file OBY-29-1363-s001.docx]

# Urbanization in Peru is inversely associated with double burden of malnutrition: pooled analysis of 92,841 mother-child pairs

Daniel Mendoza-Quispe^1^, Akram Hernández-Vásquez^1^, J. Jaime Miranda^1,2^, Cecilia Anza-Ramirez^1^, Rodrigo M. Carrillo-Larco^1,3^, Marco Pomati^4^, Shailen Nandy^4^, Antonio Bernabe-Ortiz^1*^

^1^ CRONICAS Center of Excellence in Chronic Diseases, Universidad Peruana Cayetano Heredia, Lima, Peru.

^2^ School of Medicine, Universidad Peruana Cayetano Heredia, Lima, Peru.

^3^ Department of Epidemiology and Biostatistics, School of Public Health, Imperial College London, London, UK.

^4^ School of Social Sciences, Cardiff University, Glamorgan Building, King Edward VII Avenue, Cardiff, Wales CF24 3PG, UK.

## Corresponding author

Antonio Bernabe-Ortiz, MD, PhD

E-mail address: [Antonio.Bernabe@upch.pe](mailto:Antonio.Bernabe@upch.pe)

Full Postal address: CRONICAS Center of Excellence in Chronic Diseases, Universidad Peruana Cayetano Heredia. Av. Armendariz 445, Miraflores, Lima 18, Peru.

**Table S1. Included versus excluded participants**

| Characteristics | | Excluded | Included | Total | Difference |
| --- | --- | --- | --- | --- | --- |
|  |  | N = 8,938 (%) | N = 92,841 (%) | N = 101,779 (%) | (%) |
| **Child sex** | |  |  |  |  |
|  | Female | 4,283 (48.5) | 45,583 (48.9) | 49,866 (48.9) | 0.4 |
|  | Male | 4,655 (51.5) | 47,258 (51.1) | 51,913 (51.1) | 0.4 |
| **Child age (years)** | | |  |  |  |
|  | <2 | 1,875 (21.6) | 38,349 (41.4) | 40,224 (39.7) | 19.8 |
|  | 2-5 | 7,063 (78.4) | 54,492 (58.6) | 61,555 (60.3) | 19.8 |
| **Mother’s age (years)** | | |  |  |  |
|  | 15-24 | 1,739 (30.1) | 24,989 (25.9) | 26,728 (26.1) | 4.2 |
|  | 25-34 | 2,604 (48.9) | 43,086 (46.8) | 45,690 (46.9) | 2.1 |
|  | 35-49 | 1,096 (21.0) | 24,766 (27.3) | 25,862 (26.9) | 6.3 |
| **Mother's educational attainment** | | |  |  |  |
|  | Primary or less | 2,172 (37.4) | 29,200 (29.4) | 31,372 (29.9) | 8.0 |
|  | Secondary | 2,801 (53.5) | 55,004 (60.9) | 57,805 (60.5) | 7.4 |
|  | Superior | 464 (9.0) | 8,636 (9.7) | 9,100 (9.6) | 0.7 |
| **Socioeconomic status** | | | |  |  |
|  | Very Poor | 2,153 (20.8) | 17,818 (16.5) | 19,971 (16.8) | 4.3 |
|  | Poor | 1,718 (18.5) | 18,165 (18.6) | 19,883 (18.6) | 0.1 |
|  | Middle | 1,682 (18.7) | 18,260 (19.4) | 19,942 (19.4) | 0.7 |
|  | Rich | 1,611 (20.0) | 18,261 (20.9) | 19,872 (20.9) | 0.9 |
|  | Very Rich | 1,513 (22.0) | 18,357 (24.6) | 19,870 (24.3) | 2.6 |
| **Altitude (m.a.s.l.)** | | |  |  |  |
|  | <2500 | 6,546 (74.8) | 65,516 (74.0) | 72,062 (74.1) | 0.8 |
|  | ≥2500 | 2,366 (25.2) | 27,138 (26.0) | 29,504 (25.9) | 0.8 |
| **Urbanization level (inh/km^2^)** | | |  |  |  |
|  | 1-500 | 7,138 (68.8) | 69,746 (61.7) | 76,884 (62.3) | 7.1 |
|  | 501-1,000 | 501 (5.5) | 6,389 (6.7) | 6,890 (6.6) | 1.2 |
|  | 1,001- 2,500 | 244 (3.0) | 3,164 (3.4) | 3,408 (3.3) | 0.4 |
|  | 2,501- 5,000 | 156 (2.3) | 2,148 (3.2) | 2,304 (3.1) | 0.9 |
|  | 5,001-7,500 | 257 (4.3) | 3,179 (5.2) | 3,436 (5.2) | 0.9 |
|  | 7,501-10,000 | 226 (5.5) | 3,161 (7.2) | 3,387 (7.1) | 1.7 |
|  | 10,001-15,000 | 180 (5.4) | 2,074 (5.7) | 2,254 (5.6) | 0.3 |
|  | ≥15,001 | 166 (5.3) | 2,347 (7.0) | 2,513 (6.8) | 1.7 |
| **Child undernutrition** | |  |  |  |  |
|  | No | 6,749 (78.2) | 74,842 (82.0) | 81,591 (81.6) | 3.8 |
|  | Yes | 2,124 (21.8) | 17,999 (18.0) | 20,123 (18.4) | 3.8 |
| **Maternal overweight/obesity** | | |  |  |  |
|  | No | 1,898 (36.5) | 37,971 (40.3) | 39,869 (40.1) | 3.8 |
|  | Yes | 3,167 (63.5) | 54,870 (59.7) | 58,037 (59.9) | 3.8 |
| **Household-level DBM** | |  |  |  |  |
|  | No | 4,323 (87.2) | 84,144 (91.0) | 88,467 (90.8) | 3.8 |
|  | Yes | 682 (12.8) | 8,697 (9.0) | 9,379 (9.2) | 3.8 |
| Frequencies are unweighted, and percentages are weigthed accounting for the complex survey design.  Abbreviations: m.a.s.l. = meters above sea level; inh/km^2^ = inhabitants/km^2^; DBM = double burden of malnutrition. | | | | | |

**Table S2. Trends in the household-level DBM (2009-2016, Peru)**

| Characteristics | | 2009 | 2010 | 2011 | 2012 | 2013 | 2014 | 2015 | 2016 | Total | p for trend* | 2016-2009 difference | |
| --- | --- | --- | --- | --- | --- | --- | --- | --- | --- | --- | --- | --- | --- |
|  |  | % | % | % | % | % | % | % | % | % |  | % | p-value** |
| **Child** | |  |  |  |  |  |  |  |  |  |  |  |  |
|  | No failure | 76.1 | 77 | 80.4 | 81.4 | 82.3 | 84.7 | 84.9 | 86.5 | 81.9 | **<0.001** | **+10.4** | **<0.001** |
|  | Only wasting | 0.2 | 0.2 | 0.1 | 0.2 | 0.0 | 0.2 | 0.2 | 0.1 | 0.1 | **0.045** | -0.1 | 0.285 |
|  | Wasting & underweight | 0.2 | 0.2 | 0.1 | 0.3 | 0.2 | 0.3 | 0.5 | 0.3 | 0.3 | **<0.001** | +0.1 | 0.595 |
|  | Wasting & underweight & stunting | 0.1 | 0.3 | 0.2 | 0.2 | 0.1 | 0.1 | 0.2 | 0.2 | 0.2 | **0.046** | +0.1 | 0.129 |
|  | Underweight & stunting | 3.6 | 3.4 | 3.4 | 2.7 | 2.9 | 2.3 | 2.2 | 2.2 | 2.8 | **<0.001** | -1.4 | **<0.001** |
|  | Only stunting | 19.6 | 18.6 | 15.6 | 15.0 | 14.2 | 12.1 | 11.6 | 10.4 | 14.4 | **<0.001** | **-9.2** | **<0.001** |
|  | Only underweight | 0.2 | 0.2 | 0.3 | 0.2 | 0.3 | 0.3 | 0.3 | 0.3 | 0.3 | 0.274 | +0.1 | **0.041** |
| **Mother** | |  |  |  |  |  |  |  |  |  |  |  |  |
|  | Normal | 47.2 | 46.3 | 41.7 | 41.0 | 38.5 | 36.4 | 37.0 | 36.7 | 40.3 | **<0.001** | **-10.5** | **<0.001** |
|  | Overweight | 38.0 | 38.4 | 40.8 | 40.6 | 40.4 | 42.2 | 40.4 | 40.7 | 40.2 | **0.007** | +2.7 | **0.005** |
|  | Obesity | 14.8 | 15.4 | 17.5 | 18.4 | 21.1 | 21.4 | 22.6 | 22.6 | 19.4 | **<0.001** | **+7.8** | **<0.001** |
| **DBM** | |  |  |  |  |  |  |  |  |  |  |  |  |
|  | Normal | 34.0 | 33.4 | 31.4 | 32.1 | 30.1 | 29.1 | 30.2 | 30.4 | 31.3 | **<0.001** | -3.6 | **<0.001** |
|  | Mother’s overweight/obesity only | 42.1 | 43.6 | 49.0 | 49.3 | 52.1 | 55.7 | 54.8 | 56.1 | 50.7 | **<0.001** | **+14** | **<0.001** |
|  | Child undernutrition only | 13.2 | 12.9 | 10.3 | 8.8 | 8.4 | 7.3 | 6.8 | 6.3 | 9.1 | **<0.001** | **-6.9** | **<0.001** |
|  | Double burden | 10.7 | 10.1 | 9.3 | 9.7 | 9.4 | 8.0 | 8.3 | 7.2 | 9.0 | **<0.001** | -3.5 | **<0.001** |
| * Global p-value of a poisson regression model adjusted by survey year  ** Contrasts of marginal linear predictions.  Frequencies are unweighted, and percentages are weigthed accounting for the complex survey design. P-values <0.05 and differences beyond +-5% shown in bold Abbreviations: DBM = double burden of malnutrition. | | | | | | | | | | | | | |

**Table S3. Population description by survey year (2009-2016, Peru)**

| Characteristics | | 2009 | 2010 | 2011 | 2012 | 2013 | 2014 | 2015 | 2016 | Total |
| --- | --- | --- | --- | --- | --- | --- | --- | --- | --- | --- |
|  |  | N=8,815 (%) | N=8,270 (%) | N=8,329 (%) | N=8,733 (%) | N=8,123 (%) | N=8,763 (%) | N=22,215 (%) | N=19,593 (%) | N=92,841 (%) |
| **Child sex** | |  |  |  |  |  |  |  |  |  |
|  | Female | 4,287 (48.5) | 4,165 (49.9) | 4,121 (49.2) | 4,320 (49.2) | 4,041 (49.1) | 4,261 (48.9) | 10,904 (48.8) | 9,484 (48.3) | 45,583 (48.9) |
|  | Male | 4,528 (51.5) | 4,105 (50.1) | 4,208 (50.8) | 4,413 (50.8) | 4,082 (50.9) | 4,502 (51.1) | 11,311 (51.2) | 10,109 (51.7) | 47,258 (51.1) |
| **Child age (years)** | | |  |  |  |  |  |  |  |  |
|  | <2 | 3,721 (41.6) | 3,502 (42.5) | 3,419 (40.9) | 3,631 (41.9) | 3,385 (41.6) | 3,699 (42.4) | 9,179 (41.2) | 7,813 (39.7) | 38,349 (41.4) |
|  | 2-5 | 5,094 (58.4) | 4,768 (57.5) | 4,910 (59.1) | 5,102 (58.1) | 4,738 (58.4) | 5,064 (57.6) | 13,036 (58.8) | 11,780 (60.3) | 54,492 (58.6) |
| **Mother’s age (years)** | | |  |  |  |  |  |  |  |  |
|  | 15-24 | 2,550 (26.7) | 2,328 (27.3) | 2,260 (25.7) | 2,494 (27.4) | 2,257 (26.5) | 2,378 (25.9) | 5,773 (24.5) | 4,949 (24.1) | 24,989 (25.9) |
|  | 25-34 | 4,033 (46.6) | 3,823 (46.6) | 3,856 (47.2) | 3,959 (45.7) | 3,752 (47.0) | 4,054 (46.5) | 10,363 (46.6) | 9,246 (48.0) | 43,086 (46.8) |
|  | 35-49 | 2,232 (26.7) | 2,119 (26.1) | 2,213 (27.1) | 2,280 (26.9) | 2,114 (26.5) | 2,331 (27.6) | 6,079 (28.8) | 5,398 (27.9) | 24,766 (27.3) |
| **Mother's educational attainment**^†^ | | |  |  |  |  |  |  |  |  |
|  | Primary or less | 3,566 (35.3) | 3,287 (35.3) | 3,238 (33.2) | 3,113 (31.7) | 2,753 (29.3) | 2,698 (26.1) | 5,703 (24.2) | 4,842 (23.8) | 29,200 (29.4) |
|  | Secondary | 4,749 (57.1) | 4,447 (57.6) | 4,531 (58.4) | 4,966 (60.0) | 4,690 (61.7) | 5,237 (63.4) | 14,019 (63.8) | 12,365 (63.3) | 55,004 (60.9) |
|  | Superior | 500 (7.6) | 536 (7.1) | 560 (8.4) | 654 (8.3) | 680 (9.1) | 828 (10.5) | 2,492 (12.0) | 2,386 (12.9) | 8,636 (9.7) |
| **Socioeconomic status**^†^ | | | |  |  |  |  |  |  |  |
|  | Very poor | 1,709 (15.9) | 1,648 (17.6) | 1,610 (15.7) | 1,353 (17.0) | 1,576 (15.4) | 1,729 (15.7) | 4,333 (17.0) | 3,860 (17.4) | 17,818 (16.5) |
|  | Poor | 1,763 (18.3) | 1,630 (18.8) | 1,669 (19.0) | 1,368 (18.8) | 1,632 (18.7) | 1,742 (18.2) | 4,426 (18.3) | 3,935 (18.6) | 18,165 (18.6) |
|  | Middle | 1,776 (19.5) | 1,692 (19.0) | 1,671 (19.3) | 1,338 (18.9) | 1,623 (19.6) | 1,776 (20.6) | 4,445 (19.3) | 3,939 (19.1) | 18,260 (19.4) |
|  | Rich | 1,800 (21.4) | 1,618 (20.7) | 1,698 (20.8) | 1,357 (20.7) | 1,649 (21.3) | 1,740 (21.5) | 4,473 (20.9) | 3,926 (20.4) | 18,261 (20.9) |
|  | Very rich | 1,767 (24.9) | 1,682 (23.8) | 1,681 (25.2) | 1,337 (24.6) | 1,643 (25.0) | 1,776 (23.9) | 4,538 (24.6) | 3,933 (24.5) | 18,357 (24.6) |
| **Altitude (m.a.s.l.)**^†^ | | |  |  |  |  |  |  |  |  |
|  | <2,500 | 5,745 (68.7) | 5,465 (69.7) | 5,619 (71.6) | 6,041 (72.0) | 5,732 (76.2) | 6,172 (75.7) | 16,265 (78.1) | 14,477 (77.9) | 65,516 (74.0) |
|  | ≥2,500 | 3,054 (31.3) | 2,791 (30.3) | 2,691 (28.4) | 2,672 (28.0) | 2,375 (23.8) | 2,566 (24.3) | 5,915 (21.9) | 5,074 (22.1) | 27,138 (26.0) |
| **Urbanization level (inh/km^2^)**^†^ | | |  |  |  |  |  |  |  |  |
|  | 1-500 | 7,188 (66.0) | 6,721 (65.3) | 6,744 (63.1) | 6,710 (63.6) | 6,240 (61.3) | 6,479 (59.9) | 15,617 (57.2) | 14,047 (59.2) | 69,746 (61.7) |
|  | 501-1,000 | 439 (5.6) | 444 (5.9) | 478 (6.8) | 624 (7.4) | 572 (6.3) | 598 (7.0) | 1,800 (7.9) | 1,434 (6.2) | 6,389 (6.7) |
|  | 1,001-2,500 | 247 (2.9) | 228 (3.1) | 186 (2.9) | 262 (3.6) | 210 (2.9) | 246 (3.7) | 893 (3.5) | 892 (4.2) | 3,164 (3.4) |
|  | 2,501-5,000 | 204 (3.9) | 177 (3.7) | 212 (5.2) | 232 (3.0) | 216 (3.0) | 248 (3.2) | 492 (2.6) | 367 (1.7) | 2,148 (3.2) |
|  | 5,001-7,500 | 248 (7.3) | 204 (6.6) | 171 (4.4) | 246 (4.7) | 208 (3.8) | 312 (4.2) | 918 (5.2) | 872 (5.8) | 3,179 (5.2) |
|  | 7,501-10,000 | 114 (3.0) | 128 (3.7) | 174 (6.2) | 196 (5.7) | 245 (9.2) | 369 (9.0) | 1,074 (10.1) | 861 (9.2) | 3,161 (7.2) |
|  | 10,001-15,000 | 131 (4.3) | 160 (5.6) | 166 (5.0) | 246 (6.8) | 173 (5.5) | 200 (5.4) | 515 (6.1) | 483 (6.2) | 2,074 (5.7) |
|  | ≥15,001 | 185 (7.0) | 159 (6.2) | 169 (6.5) | 174 (5.2) | 205 (8.0) | 251 (7.6) | 650 (7.5) | 554 (7.5) | 2,347 (7.0) |
| **Child undernutrition** | |  |  |  |  |  |  |  |  |  |
|  | No | 6,432 (76.1) | 6,167 (77.0) | 6,371 (80.4) | 6,902 (81.4) | 6,438 (82.3) | 7,172 (84.7) | 18,590 (84.9) | 16,770 (86.5) | 74,842 (82.0) |
|  | Yes | 2,383 (23.9) | 2,103 (23.0) | 1,958 (19.6) | 1,831 (18.6) | 1,685 (17.7) | 1,591 (15.3) | 3,625 (15.1) | 2,823 (13.5) | 17,999 (18.0) |
| **Maternal overweight/obesity** | | |  |  |  |  |  |  |  |  |
|  | No | 4,373 (47.2) | 3,948 (46.3) | 3,733 (41.7) | 3,725 (41.0) | 3,273 (38.5) | 3,423 (36.4) | 8,311 (37.0) | 7,185 (36.7) | 37,971 (40.3) |
|  | Yes | 4,442 (52.8) | 4,322 (53.7) | 4,596 (58.3) | 5,008 (59.0) | 4,850 (61.5) | 5,340 (63.6) | 13,904 (63.0) | 12,408 (63.3) | 54,870 (59.7) |
| **Household-level DBM** | |  |  |  |  |  |  |  |  |  |
|  | No | 7,820 (89.3) | 7,387 (89.9) | 7,444 (90.7) | 7,827 (90.3) | 7,262 (90.6) | 8,011 (92.0) | 20,267 (91.7) | 18,126 (92.8) | 84,144 (91.0) |
|  | Yes | 995 (10.7) | 883 (10.1) | 885 (9.3) | 906 (9.7) | 861 (9.4) | 752 (8.0) | 1,948 (8.3) | 1,467 (7.2) | 8,697 (9.0) |
| Frequencies are unweighted, and percentages are weigthed accounting for the complex survey design.  †Some columns may no add due to missing data.  Abbreviations: m.a.s.l. = meters above sea level; inh/km^2^ = inhabitants/km^2^; DBM = double burden of malnutrition. | | | | | | | | | | |
|  | | | | | | | | | | |

**Table S4. Bivariate analysis of factors associated with the household-level DBM**

| Characteristics | | DBM | | p* |
| --- | --- | --- | --- | --- |
|  |  | No | Yes |  |
|  |  | N=84,144 (%) | N=8,697 (%) |  |
| **Child sex** | |  |  | **0.038** |
|  | Female | 41,535 (91.3) | 4,048 (8.7) |  |
|  | Male | 42,609 (90.8) | 4,649 (9.2) |  |
| **Child age (years)** | |  |  | **<0.001** |
|  | <2 | 35,070 (91.6) | 3,279 (8.4) |  |
|  | 2-5 | 49,074 (90.6) | 5,418 (9.4) |  |
| **Mother’s age (years)** | | |  | **<0.001** |
|  | 15-24 | 23,311 (93.2) | 1,678 (6.8) |  |
|  | 25-34 | 39,109 (91.2) | 3,977 (8.8) |  |
|  | 35-49 | 21,724 (88.7) | 3,042 (11.3) |  |
| **Mother’s educational attainment** | | |  | **<0.001** |
|  | Primary or less | 24,445 (83.1) | 4,755 (16.9) |  |
|  | Secondary | 51,326 (93.8) | 3,678 (6.2) |  |
|  | Superior | 8,372 (97.4) | 264 (2.6) |  |
| **Socioeconomic status** | | |  | **<0.001** |
|  | Very poor | 15,434 (85.9) | 2,384 (14.1) |  |
|  | Poor | 16,164 (88.6) | 2,001 (11.4) |  |
|  | Middle | 16,527 (90.6) | 1,733 (9.4) |  |
|  | Rich | 16,907 (93.2) | 1,354 (6.8) |  |
|  | Very rich | 17,427 (95.5) | 930 (4.5) |  |
| **Altitude (m.a.s.l.)** | |  |  | **<0.001** |
|  | <2,500 | 60,675 (92.9) | 4,841 (7.1) |  |
|  | ≥2,500 | 23,303 (85.7) | 3,835 (14.3) |  |
| **Urbanization level (inh/km^2^)** | | | | **<0.001** |
|  | 1-500 | 62,299 (88.5) | 7,447 (11.5) |  |
|  | 501-1,000 | 5,985 (92.4) | 404 (7.6) |  |
|  | 1,001- 2,500 | 2,987 (94.7) | 177 (5.3) |  |
|  | 2,501- 5,000 | 1,983 (95.4) | 165 (4.6) |  |
|  | 5,001-7,500 | 3,021 (94.8) | 158 (5.2) |  |
|  | 7,501-10,000 | 3,051 (96.3) | 110 (3.7) |  |
|  | 10,001-15,000 | 1,978 (95.5) | 96 (4.5) |  |
|  | ≥15,001 | 2,266 (96.7) | 81 (3.3) |  |
| *Chi-squared test.  Frequencies are unweighted, and percentages are weigthed accounting for the complex survey design.  Abbreviations: m.a.s.l. = meters above sea level; inh/km^2^ = inhabitants/km^2^; DBM = double burden of malnutrition. | | | | |

**Table S5. Short height and nutritional status of children and mothers, with urban-rural stratification**

| **Child undernutrition** | | Crude prevalence | | Crude model* | | Adjusted model* | |
| --- | --- | --- | --- | --- | --- | --- | --- |
|  |  | % | 95% CI | PR | 95% CI | PR | 95% CI |
| **Mother with short height from rural areas**** | | | |  |  |  |  |
|  | No | 29.3 | 15.0 - 15.9 | Ref. |  | Ref. |  |
|  | Yes | 55.7 | 38.9 - 41.9 | **1.90** | **1.83 - 1.98** | **1.72** | **1.65 - 1.79** |
| **Mother with short height from urban areas**** | | | |  |  |  |  |
|  | No | 9.4 | 9.0 - 9.8 | Ref. |  | Ref. |  |
|  | Yes | 27.1 | 25.1 - 29.0 | **2.88** | **2.67 - 3.12** | **2.12** | **1.96 - 2.29** |
| **Mother with overweight/obesity** | | Crude prevalence | | Crude model* | | Adjusted model* | |
|  |  | % | 95% CI | PR | 95% CI | PR | 95% CI |
| **Mother with short height from rural areas**** | | | |  |  |  |  |
|  | No | 49.9 | 48.8 - 50.9 | Ref. |  | Ref. |  |
|  | Yes | 50.5 | 48.3 - 52.7 | 1.01 | 0.97 - 1.06 | 1.03 | 0.99 - 1.08 |
| **Mother with short height from urban areas**** | | | |  |  |  |  |
|  | No | 64.1 | 63.4 - 64.7 | Ref. |  | Ref. |  |
|  | Yes | 66.6 | 64.5 - 68.7 | **1.04** | **1.01 - 1.07** | 1.02 | 0.99 - 1.06 |
| *Poisson log generalized linear models, accounting for the complex survey design.  **The analysis was stratified by urban and rural areas.  Estimates with p-values <0.05 shown in bold.  Model adjusted by urbanization level, sex and age of child, age and educational attainment of mother, short height of mother, socioeconomic status, altitude and survey year.  Abbreviations: PR = prevalence ratio; 95% CI = 95% confidence interval; ref = reference. | | | | | | | |
